# Temporal constraints on the potential role of fry odors as cues of past reproductive success for spawning lake trout

**DOI:** 10.1002/ece3.3546

**Published:** 2017-10-24

**Authors:** Tyler J. Buchinger, J. Ellen Marsden, Thomas R. Binder, Mar Huertas, Ugo Bussy, Ke Li, James E. Hanson, Charles C. Krueger, Weiming Li, Nicholas S. Johnson

**Affiliations:** ^1^ Department of Fisheries and Wildlife Michigan State University East Lansing MI USA; ^2^ Rubenstein Ecosystem Science Laboratory Rubenstein School of Environment and Natural Resources University of Vermont Burlington VT USA; ^3^ Department of Chemistry and Biochemistry Seton Hall University South Orange NJ USA; ^4^ U.S. Geological Survey Hammond Bay Biological Station Millersburg MI USA; ^5^ Present address: Department of Biology Texas State University San Marcos TX USA

**Keywords:** conspecific cues, habitat selection, olfaction, *Salvelinus namaycush*

## Abstract

Deciding where to reproduce is a major challenge for most animals. Many select habitats based upon cues of successful reproduction by conspecifics, such as the presence of offspring from past reproductive events. For example, some fishes select spawning habitat following odors released by juveniles whose rearing habitat overlaps with spawning habitat. However, juveniles may emigrate before adults begin to search for spawning habitat; hence, the efficacy of juvenile cues could be constrained by degradation or dissipation rates. In lake trout (*Salvelinus namaycush*), odors deposited by the previous year's offspring have been hypothesized to guide adults to spawning reefs. However, in most extant populations, lake trout fry emigrate from spawning reefs during the spring and adults spawn during the fall. Therefore, we postulated that the role of fry odors in guiding habitat selection might be constrained by the time between fry emigration and adult spawning. Time course chemical, physiological, and behavioral assays indicated that the odors deposited by fry likely degrade or dissipate before adults select spawning habitats. Furthermore, fry feces did not attract wild lake trout to constructed spawning reefs in Lake Huron. Taken together, our results indicate fry odors are unlikely to act as cues for lake trout searching for spawning reefs in populations whose juveniles emigrate before the spawning season, and underscore the importance of environmental constraints on social cues.

## 1 INTRODUCTION

Cues that indicate successful reproduction by conspecifics often guide selection of reproductive habitats (Danchin, Giraldeau, Valone, & Wagner, 2004; Doligez, Danchin, & Clobert, 2002). Choosing habitat randomly (Dale & Slagsvold, 1990) or through direct assessment of physical and environmental attributes (Clark & Shutler, 1999) can be costly (Morris, 1992) and less effective than returning to a previously used breeding site (Switzer, 1993) or using conspecific cues of past reproductive success (Boulinier & Danchin, 1997). For example, comparing the quality of multiple sites consumes time and energy, and direct evaluation provides information that may not be consistent across time. Cues of conspecific success can come from other reproductive adults (Deutsch & Nefdt, 1992) or offspring from past reproductive events (Doligez et al., 2002), and originate directly from current residents (Doligez et al., 2002), persist from conspecifics that are no longer present (Boulinier & Danchin, 1997), or be learned during previous observations (Schjørring, Gregersen, & Bregnballe, 1999). Many known cues of conspecific reproductive success are visual (Valone, 2007), but chemical cues can be particularly informative for habitat selection in some animals (Wyatt, 2014).

Many migratory fishes navigate to spawning habitat using chemical cues (Bett & Hinch, 2016). Chemical cues can function over large temporal and spatial scales (Wyatt, 2014) and, as a result, are suited to guide migrations. In some species, chemical cues released by stream‐resident conspecifics guide navigation to spawning habitat. For example, sea lamprey (*Petromyzon marinus*) migrates into spawning streams following the odor of stream‐resident larvae (Teeter, 1980), which indicates success of offspring in a stream (Polkinghorne, Olson, Gallaher, & Sorensen, 2001; Wagner, Twohey, & Fine, 2009). Larval sea lamprey resides immediately downstream of spawning habitat for several years (Dawson, Quintella, Almeida, Treble, & Jolley, 2015); hence, cues that guide sea lamprey are constantly replenished and present when adults immigrate to spawn. In many species, however, juveniles emigrate before reproductive adults search for and select spawning habitats. A disconnect between when juveniles and reproductive adults occupy spawning habitat may constrain the efficacy of juvenile‐released conspecific cues. Alternatively, conspecific cues may persist at spawning habitats after juveniles emigrate until adults return to reproduce.

**Figure 1:**
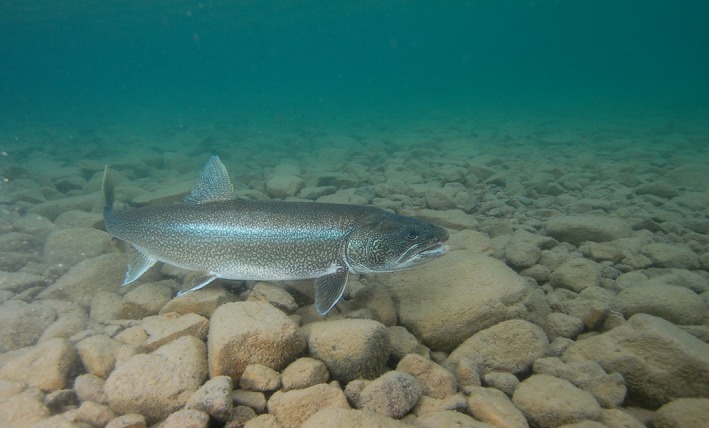
Adult lake trout (*Salvelinus namaycush*) in Great Bear Lake, Canada (photo credit: A. Muir and P. Vecsei)

Residual chemical cues deposited by juveniles have been hypothesized to guide habitat selection by spawning lake trout, *Salvelinus namaycush* (Figure 1; Foster, 1985). Most populations of lake trout occupy lake environments and spawn over nearshore reefs during autumn (Muir, Blackie, Marsden, & Krueger, 2012). Foster (1985) hypothesized that fecal bile acids and other waste products deposited by fry on productive reefs act as cues of past reproductive success for spawning adults searching for suitable habitat. Indeed, closely related Arctic char (*S. alpinus*) likely select spawning streams using bile acids released via feces by stream‐resident juveniles (Nordeng, 1971; Selset & Døving, 1980). Unlike fishes whose occupancy of habitat used for juvenile rearing and adult spawning overlaps, lake trout juveniles are absent from spawning habitat for several months before adults return (Deroche, 1969; Martin, 1957). For example, Deroche (1969) reported that in Thompson Lake, Maine most eggs hatched in March, fry left the spawning site by early May, and adult spawning began in middle October. Notably, the timing of development, departure from the reef, and adult spawning likely differ among populations. Regardless, the time interval between juvenile emigration and adult spawning may constrain the efficacy of juvenile odors as indicators of high quality spawning habitat.

We evaluated the hypothesis that fry odors act as cues of past reproductive success during selection of spawning habitat by lake trout. Although previous experiments indicated that juveniles release potent odors (Zhang, Brown, & Hara, 2001) that direct where lake trout spawn in laboratory assays (Foster, 1985), the role of fry odors as a conspecific chemical cue has not been determined in the ecological context of natural lake trout reproduction. We tested the potential role of fry odors as chemical cues for spawning lake trout using (1) chemical, physiological, and behavioral assays to determine the persistence and attractiveness of lake trout fry odors over an ecologically relevant temporal scale, and (2) a field experiment that tested whether adult lake trout preferentially selected spawning habitat treated with fry feces over similar habitats that were not. Specifically, we predicted that if fry odors act as cues of past reproductive success, (1) the odor should persist from when fry disperse from the reef to when adults return and (2) spawning lake trout should visit reefs treated with fry feces in higher proportions and for longer durations of time compared to reefs not treated with fry feces.

## 2 METHODS

### 2.1 Persistence of fry odors through the spawning season

#### 2.1.1 Overview

We used chemical analyses, electro‐olfactogram (EOG) recordings, and behavioral assays to evaluate the persistence and attractiveness of fry odors over an ecologically relevant temporal scale. First, we held lake trout fry in aquaria containing substratum and supplied with ambient‐temperature Lake Huron water for 4 months, at which point we removed the fish and sampled water from the tanks every 2 weeks for 4 months. We then determined the olfactory potency of the samples collected across the time series using EOG recordings and bile acid concentrations across the time series using ultrahigh‐performance liquid chromatography with tandem mass spectrometry (UHPLC‐MS/MS). Second, we used behavioral assays to evaluate attraction of spawning lake trout to the effluent of tanks that had previously held fry. Lake trout fry used for odor collection had already began exogenous feeding and, for simplicity, all age‐0 juveniles are referred to as fry. All procedures outlined in this study were approved by the Michigan State University Animal Use and Care Committee (AUF Numbers 08/12‐148‐00 and 09/15‐135‐00).

#### 2.1.2 Odor collection

In 2013, we collected fry odors to evaluate their persistence until the spawning season using chemical analyses and EOG recordings. In early April, 1,200 (~0.5 g each) age‐0 Seneca strain lake trout from the U.S. Fish and Wildlife Service Jordan River National Fish Hatchery were transported to the U.S. Geological Survey Hammond Bay Biological Station (HBBS). At HBBS, lake trout were held in three glass aquaria (76 × 33 × 33 cm, 75 L, ~400 lake trout each) alongside a fourth aquarium maintained as a control with no fish. Each held mixed‐type, medium‐sized gravel (~5–10 cm) substrate collected from the beach near HBBS. Ambient (1.1–18.4°C) Lake Huron water flowed into aquariums at a rate of approximately 100 ml/min, and flowed out through a standpipe. Maintaining water flowing through the aquariums was part of our effort to match natural conditions, as diffusion and currents would slowly dilute fry odors in lakes. Approximately 10 g Rangen trout grower food pellets (www.rangen.com) were added to all four aquaria. Mortalities were removed daily, but the aquaria were not cleaned over the duration of the experimental period to allow fry odors to accumulate. By definition, the control tank ended with more uneaten food compared to the tank that held fry. Although the difference in the quantity of uneaten food in the fry and control tanks was not ideal, no other controls seemed appropriate. Furthermore, the use of a control tank with food likely made for a more conservative experiment as the olfactory potency of the fry tanks had a smaller contribution of food odor. Mortality was minimal except during a brief period of gas supersaturation in June when approximately 25% of the fish died. All fish were removed in early August, at which point each tank held approximately 300 fish (1.03 ± 0.05 g, 4.79 ± 0.09 cm, *n* = 30, mean ± *SE*). Total weights of fish in each tank were 248, 187, and 307 g. After removing the fry, we maintained the aquaria through December. Water was sampled from each aquarium immediately after removing the fish, and over a time course of 2, 4, 6, 8, 10, 14, and 16 weeks after removing the fish. At each time‐point, we disturbed the water to mix potential odors that settled into interstitial spaces, collected 1 L of water from each aquarium, and stored the samples at less than −20°C for quantification of bile acids and EOG recordings. The fry odors presumably contained all materials released by the fish, including urine, feces, and mucus as well as unconsumed food.

In 2016, we collected fry odors to evaluate their persistence until the spawning season using behavioral assays. We held 900 lake trout fry in one 200‐L tank alongside a second 200‐L tank that held no fish, but otherwise used the same methods described above. Few mortalities occurred from April through August when we moved all fish to a third tank. After high mortality in September, again due to gas supersaturation, we received a new group of approximately 250 (~13 g each) lake trout from the same hatchery, strain, and year class, which would provide positive control odor treatments for subsequent behavioral assays (described below).

#### 2.1.3 Quantification of putative chemical cues

We quantified 16 bile acids hypothesized to guide adult lake trout to spawning reefs (Zhang et al., 2001) to determine their presence as odorants during the spawning season (Li et al., 2015). We added an internal standard of 10 ng deuterated cholic acid and taurocholic acid (CA‐d_4_ and TCA‐d_4_) to a 10 ml subsample of the 1 L samples collected from aquaria over 16 weeks, as described above. Each sample was freeze‐dried using a CentriVap Cold Trap with CentriVap Concentrator (Labconco, MO) and reconstituted in 100 μl of methanol and water (1:1, v:v). Reconstituted samples were subjected to UHPLC‐MS/MS (Waters Acquity ultra‐performance liquid chromatography system, Waters, Milford, MA; Micromass Quattro Premier XE tandem quadruple mass spectrometer, Waters, Manchester, UK) using described methods (Li et al., 2015).

#### 2.1.4 Olfactory responses to fry odors

We evaluated changes in residual fry odors that might occur between the times fry emerge from the reef and adults return to spawn using EOG recordings. EOG recordings determined the olfactory responses to samples collected from aquaria over 16 weeks and using previously described protocols (Buchinger et al., 2017; Zhang et al., 2001). Following the methods of previous studies on lake trout olfaction, we used juvenile (2 year; 2014 Seneca Lake strain) lake trout as a proxy for adults (Zhang et al., 2001). Notably, olfactory detection of conspecific odors in closely related Arctic char is not affected by life stage (Sveinsson & Hara, 2000). Briefly, lake trout were anesthetized using 3‐aminobenzoic acid ethyl ester (50 mg/L; MS222; Sigma), immobilized with an intramuscular injection of gallamine triethiodide (1 mg/kg; Sigma), and secured in a plexiglass trough with their gills continuously perfused with aerated water. A stimulus electrode was placed between olfactory lamellae and a reference electrode placed on the skin. Electrical signals were filtered and amplified using a NeuroLog filter and preamplifier (Digitimer Ltd., Hertfordshire, England), integrated using an Axon Instruments Digidata system 1550 (Molecular Devices, CA, USA) and stored on a computer with Axoscope software 10.5. Stimuli tested in EOG recordings were 100 ml subsample of fry odor that was freeze‐dried using a FreeZone Plus Freeze Dry system with a FreeZone Bulk Tray Dryer (Labconco, MO, USA), and reconstituted in 1 ml 50% methanol (v:v). We mixed aliquots of fry odors from each of the three tanks to create an average odor.

Electro‐olfactogram recordings were used to compare the olfactory potency and mechanisms of detection for fry and control odors throughout the time course. Individual fish were used in recordings once, and all data were treated as repeated measures because we recorded the responses of an individual all treatments within an experiment. (1) The difference between the magnitude of response to fry and control odors was compared across the eight sampling periods. Stimuli were prepared by diluting 100 μl of the odor in 10 ml well water to achieve the original concentration of the sample in aquariums. We normalized the responses to stimuli by subtracting the response to a negative control (odor‐free well water) and dividing by the response to a positive control (l‐Serine at 1 × 10^**−**5^ mol/L)(Zhang et al., 2001). Paired *t*‐tests were used to evaluate differences in individuals’ responses to fry and control odors within each time‐point (α = 0.05; *n* = 6). Although the objective was to determine changes in the olfactory potency of fry odors across the time series, comparisons to the control were needed to (a) confirm that the initial fry odor was not only due to uneaten food, and (b) determine whether the residual fry odors remain detectable across the time series, not just whether their potency decrease across time. Although the comparisons between fry odors and control odors were most important for testing our hypothesis, we also used mixed‐effect linear models with a fixed effect of time and random effect of fish compared to null models using likelihood ratio tests to evaluate the change in olfactory potency of odors across time using the R package *lme4*. (2) We compared the dilution‐response relationships for fry and control odors at 0, 8, and 16 weeks postfry removal. The highest dilution at which responses to a stimulus was significantly larger than responses to the control (paired *t*‐test, α = 0.05; *n* = 6) was considered to be the electrophysiological threshold of detection (Siefkes & Li, 2004). (3) We used cross‐adaptation experiments (Caprio & Byrd, 1984) to determine if fry odors were distinct from control odors at 0, 8, and 16 weeks postfry removal. Cross‐adaptation experiments began with recording responses to the adapting stimuli at concentrations that elicited responses approximately equipotent to one another. Second, the olfactory epithelium of a fish saturated with odor A was exposed to (1) 2 × the odor A (self‐adapted control; SAC) and (2) 1 × odor A + 1 × odor B (Mix) were recorded. The responses to the SAC and the Mix were normalized by the response to the adapting stimuli, and evaluated for differences using paired *t*‐tests (α = 0.05; n = 4). A difference between the response to the SAC and the Mix indicates fish detects the odors with separate olfactory mechanisms, and the odors are therefore different from one another.

#### 2.1.5 Behavioral responses to fry odors in the laboratory

We used laboratory assays to determine behavioral responses of spawning lake trout to residual odor of lake trout fry. Behavioral assays followed established protocols (Buchinger, Li, & Johnson, 2015). Briefly, side‐by‐side artificial reefs were constructed using mixed‐type rocks (10–20 cm) from the beach near HBBS in two replicate flumes. A cement block partition separated the reefs, and a horizontal Passive Integrated Transponder (PIT) antenna surrounded each reef. The experimental area was 12.5 m × 1.85 m × 0.6 m, artificial reefs were 1.5 m × 0.85 m × 0.13 m, and water velocity was 0.014 m/s. Lake trout used in behavioral assays were age 10 spermiating males of the Seneca Lake strain (3.71 ± 0.14 kg, 71.16 ± 1.03 cm, n = 24, mean ± *SE*). Males were used in behavioral assays because they are the first to arrive at spawning reefs and are believed to select sites (Muir et al., 2012). The U.S. Fish and Wildlife Service Sullivan Creek National Fish Hatchery transported the fish to HBBS on 17 October 2016. We immediately implanted 23 mm PIT tags into the abdomen of each fish via a small incision, and used them in experiments between 29 October 2016 and 16 November 2016. Each trial consisted of four fish, which were acclimated in an enclosure downstream of the artificial reefs for at least 18 hr prior to the experiment. Odor application began 1 hr prior to sunset. At sunset, we released the fish and allowed them to swim freely throughout the entire experimental arena for 5 hr. Fish were directed downstream away from the artificial reefs between trials. The behavioral arenas were not drained and cleaned between trials due to their large size and our effort to reduce handling stress by leaving fish in arenas between trials. However, water flowed through the arenas for the 18 hr between trials and likely flushed any residual odors from previous trials. Each group of four fish was sequentially cycled through all treatments, one per night, before being exchanged with a different group of four fish. Six groups of fish were tested in each of the treatments.

Each experimental group was subjected to three treatments, which consisted of applying water from one of two sources onto each reef. The treatments were as follows: (1) water from the 200‐L tank that held lake trout fry from April to August versus water from the 200‐L tank that received food from April to August but held no fish, (2) water from a 200‐L tank holding lake trout fry versus water from a 200‐L tank that received trout food but held no fish, and (3) water from Lake Huron on both sides to confirm no bias for one side of the flume. The inflow of each tank was 1000 ml/min. Odors that may have settled were resuspended by pouring 1000 ml of Lake Huron water into the tank prior to the start of odor application. Odor treatments were applied to artificial reefs at 500 ml/min using peristaltic pumps. The remaining 500 ml/min of effluent from each tank either went into a drain or was used as an odor treatment in the second flume. The order in which treatments were applied was alternated across groups. Differences in the time lake trout spent on the control vs. odor reef within each treatment were evaluated using paired *t*‐tests (α = 0.05). The treatments tested provided a positive control (fresh fry odor), a negative control (no odor), and the focal treatment (residual fry odor). Each was compared only against the adjacent reef treated with no odor and not to the other treatments. Determining whether fresh fry odor was more, or less, attractive than residual fry odor would not have been a direct test of our hypothesis. Rather, a direct test of whether residual fry odor remained attractive across time required only a comparison of time spent on the reef treated with the residual fry odor versus the time spent on the reef treated with no odor. For these comparisons, simple paired *t*‐tests were a direct and robust statistical test to determine if lake trout responded to each treatment. Statistical analyses were conducted by individual (*n* = 24) and by group (*n* = 6).

### 2.2 Behavioral responses of spawning adults to fry odors in a natural environment

#### 2.2.1 Overview

A field test was used to corroborate the results of laboratory tests. The many examples of fish responding differently to chemical cues in laboratory experiments and natural environments made a field test an important complement to laboratory tests (Johnson & Li, 2010), regardless of the results of the laboratory tests. To evaluate attraction of lake trout to fry odors in a natural setting, we treated recently constructed artificial reefs with odors collected from fry and monitored attraction to treated reefs using acoustic telemetry.

#### 2.2.2 Study area

We evaluated adult lake trout attraction to the odor of fry feces using constructed spawning reefs in Thunder Bay, Lake Huron, near Alpena MI. Thunder Bay contains two historically important but now degraded spawning reefs (Cement reef; CEM, Cement Kiln Dust reef; CKD), one spawning reef that supports most of the current lake trout spawning activity (East Reef; Marsden et al., 2016), and 29 reefs that were constructed between 2010 and 2011 as mitigation for the degraded reefs. Four reefs were constructed in 2010 and were approximately 9 m in diameter at the base, 2.4 m across at the top, and 3 m high (Reefs B, D, E, G). The remaining twenty‐four reefs were constructed in 2011 in two parallel lines extending from the two degraded reefs. The reefs were 23 m long × 7 m wide at the base and 18 × 2.4 m at the top (CEM 1–12 and CKD 1–12). The design of the constructed reefs allowed for replicated experiments using reef treatments. We used a subset of eight of the 29 constructed reefs to evaluate selection of reefs via fecal odors. Marsden et al. (2016) describe additional details of the Thunder Bay reef designs.

#### 2.2.3 Treatments

We treated a subset of the constructed reefs with the odor of feces collected from lake trout fry. The fry was of Seneca, Lewis, and Apostle Island strains and was held at the U.S. Fish and Wildlife Jordan River National Fish Hatchery. Fecal odors were waste solids siphoned off the bottom of tanks holding lake trout fry, and presumably consisted primarily of feces, along with unconsumed food. We tested feces rather than whole odor from fry because previous studies suggested the putative chemical cues were released via feces (Foster, 1985; Selset & Døving, 1980). The reefs received feces treatments via two methods: (1) slow‐release polyethylene glycol (PEG) emitters and (2) frozen blocks of raw fry feces.

We treated the four small constructed reefs with PEG emitters impregnated with bile acids extracted from either fry feces or food pellets (control for food waste in fry feces). Feces for the PEG emitters were collected in April and May 2012. We freeze‐dried 20 L of aqueous fry feces to 1.1 kg, estimated to be roughly equivalent to that produced by 5,000 fry over two months (4 g feces/kg fish per day; Clark, Harman, & Forster, 1985). Using an established method (Li et al., 2015), we extracted bile acids from fry feces and an equal weight of food pellets (BioVita Starter #0, Bio‐Oregon www.bio‐oregon.com, and Fry crumble #1, Skretting www.skretting.us). Dry feces or food was resuspended in 3 L of ethanol, sonicated, refluxed, centrifuged, the supernatant removed, and the process repeated. The pellet was resuspended a third time in 3 L chloroform/methanol (1:1, v/v), refluxed, centrifuged, and the supernatant removed. The pellet was resuspended again in 3 L of chloroform/methanol (1:1, v/v), centrifuged, and the supernatant removed. Lastly, the pooled supernatant was evaporated and reconstituted in 2 L ethanol. Triplicate 1 ml samples were dried and used to determine the concentrations of bile acids (reported by Li et al., 2015). We mixed the extracted bile acids into eight PEG emitters (per treatment) designed to release bile acids slowly over 15 days. We poured molten polymer containing the treatment (feces extract or control) into a 250‐ml plastic container, placed the 250‐ml container open‐end down into a 950‐ml container, and poured molten polymer into the 950‐ml container. We continued the process with 2.5‐ and 5‐L containers. The final 5‐L container was fitted with a snap‐on lid, which was further secured with four zip ties through holes opened in the lid and sides of the container to allow water flow through the emitter. The emitters were 22.8 × 16.5 cm, and weighed 4.94 ± 0.05 (feces), and 5.35 ± 0.06 (food control; mean ± *SE*). The design of emitters allowed water to dissolve slowly through layers of polymer over 15 days. The emitters were encased in metal mesh and attached to a cement block with a steel cable. We deployed two emitters on each of the four small reefs on 12 October 2012, and two more on 23 October 2012. Reefs B and G received feces treatments and Reefs D and E received control treatments. The reefs with the same treatment were a minimum of 650 m apart, and the closest distance between control and treated reefs was 120 m.

We treated reefs CEM‐2 and CKD‐10 with frozen blocks of fry feces. Waste solids from hatchery tanks that held fry were siphoned, as before, and frozen in 4‐L blocks. Frozen blocks held individually in mesh bags were placed onto spawning reefs on 14 August and 16 October 2012. Reefs CEM‐2 and CKD‐10 each received five blocks of frozen fecal material on each date. We supplemented potential fry odors by also moving rocks and sediment from East Reef, where the majority of spawning occurs, to CEM‐2 and CKD‐10 on 16 October 2012. Divers filled two approximately 8‐L plastic bags with rocks and surficial sediment from East Reef and closed them with cable ties. The bags were transported by boat to each of the constructed reefs, punctured in several places, and lowered onto the reefs. We used reefs CEM‐5 and CKD‐1 as control reefs. Subsequent analysis confirmed that spawning peaked during the last week of October and first week of November (Marsden et al., 2016); hence, all odor treatments were applied prior to and during the spawning season.

#### 2.2.4 Acoustic telemetry

An array of acoustic telemetry receivers monitored attraction of tagged lake trout to reefs baited with feces. The same array and fish were a part of a larger study on lake trout reproductive behavior with additional details provided by Marsden et al. (2016). A twenty‐seven receiver (VEMCO VR2W receivers; VEMCO, Halifax, N.S, Canada) VEMCO Positioning System (VPS) array was deployed from 26 September 2012 to 27 November 2012, allowed two‐dimensional positioning of each fish. The tags (VEMCO V16‐4H transmitters; 68 mm long, 24 g in air; VEMCO, Halifax, N.S., Canada) transmitted a unique code at random intervals between 170 and 310 s. The Michigan Department of Natural Resources collected lake trout with gillnets set on East Reef. We considered using fish to which Thunder Bay was a novel site to eliminate the potential confounding effect of previous experience, but felt that naïve fish would potentially leave the area without spawning whereas fish already in the area would be most likely use fry odors to evaluate the various reefs as spawning sites. We tagged 15 females (mean total length ± 1 *SD* 71.3 ± 5.2 cm) and 25 males (mean total length ± 1 *SD* 71.3 ± 9.5 cm) on 12 October 2012 (four males) and 23 October 2012 (36 fish). We anesthetized fish with 0.8 ml/L of a 10% clove oil:ethanol solution, inserted a tag through a 2–3 cm incision made in the ventral surface posterior to the pectoral girdle, and sutured the wound. Tagging was done on site, and fish were allowed to recover in floating net pens and then released 1.4 km west of CEM reefs so fish would need to pass constructed reefs to return to East Reef. Using R programming language (R Core‐Team 2014), we compared the number of acoustic telemetry positions for each tagged individual that occurred within polygons defining each reef, as described by Marsden et al. (2016).

## 3 RESULTS

### 3.1 Fry odors did not persist into the lake trout spawning season

**Figure 2:**
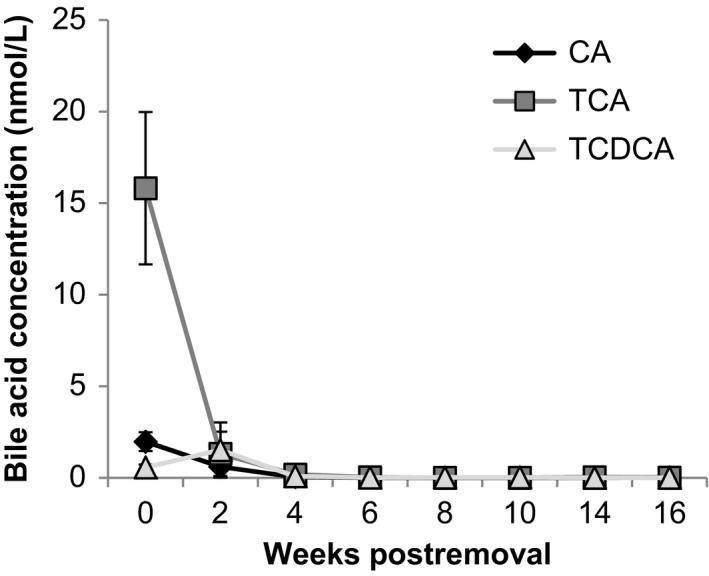
Putative bile acid chemical cues released by lake trout fry dissipated or degraded before the spawning season. Time course concentrations (nmol/L) of bile acids cholic acid (CA), taurocholic acid (TCA), and taurochenodeoxycholic acid (TCDCA) in tanks that had previously held lake trout fry. *X*‐axis indicates the number of weeks postfry removal. Of the 16 bile acids quantified (Li et al., 2015), only CA, TCA, and TCDCA were detected

Bile acids hypothesized to function as chemical cues for spawning sites either dissipated or degraded within 2 weeks of removing fish (Figure 2). Of the 16 bile acids assayed (Li et al., 2015), UHPLC‐MS/MS only cholic acid (CA), taurochenodeoxycholic acid (TCDCA), and taurocholic acid (TCA) were detected in the water samples. TCA concentrations decreased from 15.82 ± 4.16 nmol/L (mean ± *SE*) to 1.31 ± 1.21 nmol/L within 2‐week postfry removal, and continued to decrease to less than the EOG detection threshold of 1 nmol/L, (Zhang & Hara, 2009) within 4‐week postfry removal. CA concentrations decreased from 1.97 ± 0.51 nmol/L to 0.60 ± 0.59 nmol/L within 2‐week postfry removal, and continued to decrease to less than the EOG detection threshold of 0.1 nmol/L, (Zhang & Hara, 2009) within 4‐week postfry removal. TCDCA concentrations increased from 0.56 ± 0.17 nmol/L to 1.52 ± 1.50 nmol/L within 2‐week postfry removal, and then decreased to less than the EOG detection threshold of 1 nmol/L (Zhang & Hara, 2009) within 4‐week postfry removal.

**Figure 3:**
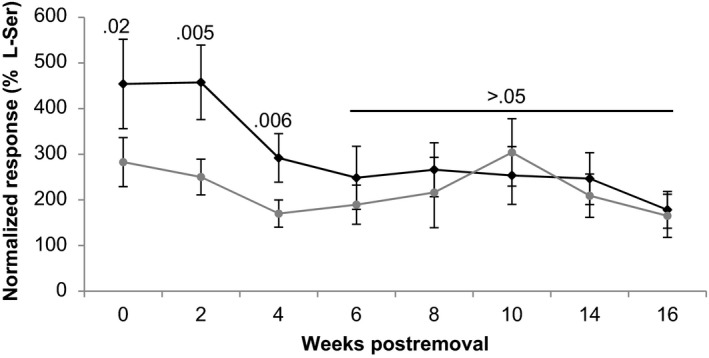
Olfactory potency of residual fry odors decreased to levels no different from controls before the spawning season. Time course olfactory responses to odors collected from tanks that had previously held fry (dark lines) or were treated as controls (light lines). Olfactory responses were measured using electro‐olfactogram recordings and standardized as a percent of the responses to 1 × 10^−5^ mol/L L‐Serine. *X*‐axis indicates the number of weeks post fry removal. *p*‐values were determined using paired *t*‐tests that compared the difference in responses to odors from the fry tanks to the control tank within a time period

**Figure 4:**
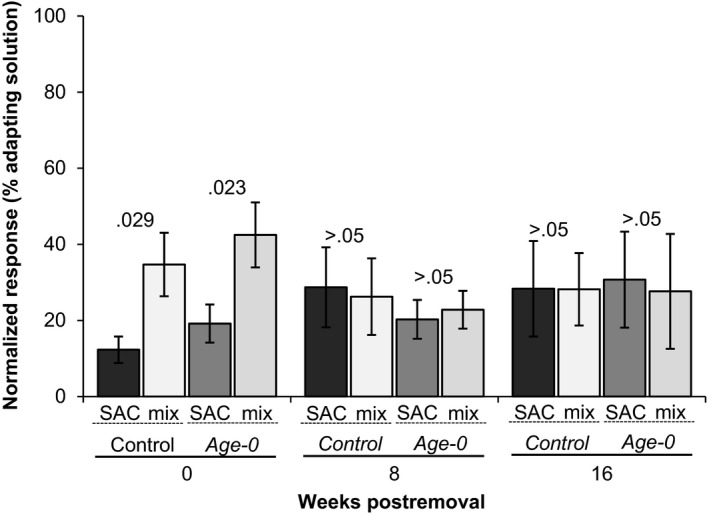
Fresh fry odors elicited responses distinct from responses to controls, but residual fry odors collected eight‐ and 16‐week postremoval did not. Cross‐adaptation experiments compared olfactory responses to control odors and fry odors when the olfactory epithelium was saturated with control or fry odors. SAC = Self‐adapted control, mix = adapted stimuli + the test stimuli. *p*‐values were determined using paired *t*‐tests to compare responses to the SAC and the mix

EOG recordings indicated that the odorants fry deposited on substrate either dissipated or degraded before the lake trout spawning season (Figure 3). Responses to 1 × 10^−5^ mol/L l‐serine and blank water were 2.23 ± 0.38 mV (*n* = 6) and 0.49 ± 0.09 mV (*n* = 6). Fry odors were initially more potent than control odors (proportion of 1 × 10^−5 ^mol/L L‐serine: fry = 4.54 ± 0.98, *n* = 6, control = 2.83 ± 0.54, *n* = 6; paired *t*‐test, *p *<* *.05), but after 4‐week postfry removal, the responses were not significantly different (paired *t*‐tests, *p *>* *.05). Mixed‐effect linear models using log‐transformed EOG response data indicated that the olfactory potency of the fry ($\chi_{1}^{2}$ = 46.48, *p *<* *.001) and the control ($\chi_{1}^{2}$ = 6.63, *p *=* *.01) odors decreased over time. The electrophysiological detection threshold for fry and control odors was a dilution of 1:10 at all time points tested (0, 8, and 16 weeks postfry removal, paired *t*‐tests, *p *>* *.05). Cross‐adaptation experiments indicated fry odors and control odors were distinct odors at 0‐week post fry removal, but not at 8‐ or 16‐week postfry removal. Adaptation to control odors did not diminish the response to fry odors, nor did adaptation to fry odors diminish the response to control odors, at 0‐week postfry removal (*n* = 4, paired *t*‐tests *p < *.05; Figure 4). The same olfactory mechanisms detected odors collected at the 8‐ and 16‐week postfry removal; adaptation to control odors diminished the response to fry odors, and vice versa (*n* = 4, paired *t*‐tests *p > *.05).

**Figure 5:**
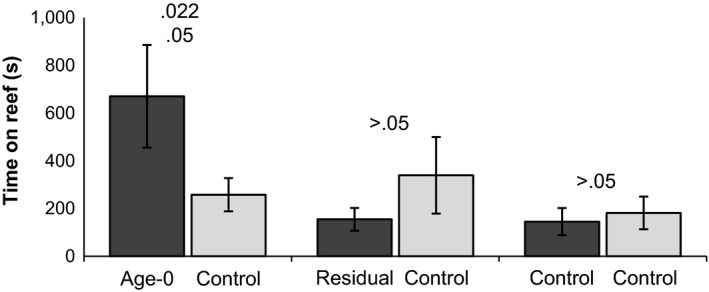
Fresh lake trout fry odors attracted spawning males in laboratory flumes but residual fry odors did not. Spawning male lake trout were presented with an artificial reef treated with a control odor versus a second artificial reef treated with (1) fresh fry odors, (2) residual fry odors, or (3) Lake Huron water. The *Y*‐axis indicates the time in seconds that individual lake trout spent on each of the adjacent reefs. P‐values were determined with paired *t*‐tests that compared the time spent on each reef after a log[x + 1] transformation (individual *p*‐value on top, group *p*‐value on bottom)

Spawning‐phase male lake trout did not spend more time on laboratory reefs treated with residual fry odors (Figure 5). Male lake trout spent similar duration of time on laboratory reefs treated with residual fry odors versus those treated with Lake Huron water (paired *t*‐tests, *p *>* *.05); nine spent more time on reefs treated with residual fry odor, 11 spent more time on reefs treated with Lake Huron water, and four did not visit either reef. Males spent a longer duration of time on reefs treated with fresh fry odors than reefs treated with Lake Huron water (paired *t*‐tests, *p* ≤ .05); sixteen spent more time on reefs treated with juvenile odor, five spent more time on reefs treated with Lake Huron water, and three did not visit either reef. We observed no bias toward the left or right reef when we applied Lake Huron water to both reefs (paired *t*‐tests, *p* > .05); nine spent more time on the left reef, 11 spent more time on the right reef, and four did not visit either reef. All time data were log [x + 1] transformed to improve normality. Analysis by individual (*n* = 24) and by group (*n* = 6) yielded the same statistical conclusions.

### 3.2 Fry feces did not increase attractiveness of spawning reefs in Lake Huron

Fry fecal odors did not attract lake trout to constructed spawning reefs. Retrieval of polymers confirmed that all polymers, except one control, dissolved properly (% remaining, feces: 11.95% ± 2.73%, control: 13.29% ± 3.61%, undissolved control: 81.24%). Detection of all 40 lake trout at a line of acoustic receivers located at the entrance to Thunder Bay (Hayden et al. 2015, http://data.glos.us/glatos) confirmed postsurgery survival of tagged fish. Twenty‐one lake trout, 17 males and three females, were detected at more than 100 unique locations within the first 2 days after tagging, confirming that they remained in the area; all of these fish were included in subsequent analyses. The acoustic telemetry array detected all but one lake trout on the natural spawning reef (East Reef), indicating that the tagging procedure did not interfere with subsequent spawning behavior. One hatchery male visited Reef E once (control polymer) and one hatchery male visited Reef G once (feces polymer). No tagged fish visited reefs B (feces polymer) and D (control polymer). Four fish (one hatchery male, two wild males, and one hatchery female) were detected on CKD‐10 (feces) for a total of 33 positions. The hatchery male also visited CEM‐2 (feces) for a total of 31 positions. Compared as positions per area of reef, there were 0.10 detections per m^2^ on CKD‐10, 0.09 on CEM‐2, an average (± *SD*) of 0.06 ± 0.04 on all of the other constructed reefs, and 205.5 on the focus of activity on East Reef (the Hot spot Reef; Marsden et al., 2016). We conducted no statistical analyses on telemetry data because there were insufficient data points for a robust analysis. Marsden et al. (2016) describe additional details of acoustic telemetry analysis and results.

## 4 DISCUSSION

Our results did not support the hypothesis that lake trout use fry odors as cues of past reproductive success (Foster, 1985). Fry released potent odors that attracted spawning‐phase lake trout in laboratory assays, but the odorants did not persist over an ecologically relevant temporal scale. Furthermore, feces applied to constructed reefs in Lake Huron did not attract wild lake trout despite previous laboratory evidence that fry feces (Foster, 1985) and juvenile odors containing feces (our results, Buchinger et al., 2015) attract spawning‐phase lake trout. We applied raw fry feces to the reefs in Lake Huron between 2 months and 2 weeks before the estimated peak spawning activity, so the potency of fecal odor was likely low during the spawning season given our laboratory results that indicated fry odors degraded or dissipated 2–4 weeks after fry removal. However, fecal odor applied via slow‐release polymers would have been constantly replenished throughout the season; hence, the odor was either not potent enough initially or not as important as other olfactory (Buchinger et al., 2015), auditory (Johnson, D. Higgs, J.E. Marsden, L. Brege, & S. Farha, in press), or visual cues. Odors from eggs and yolk‐sac larvae could be important and were not included in our experiments, but previous work with lake trout (Foster, 1985) and other species (Selset & Døving, 1980) implicate feces as the major source of juvenile chemical cues. Furthermore, the odors we collected elicited olfactory and behavioral responses, just not over the timeline that would be necessary for them to guide habitat selection. We suggest that our results do not directly conflict with previous reports on the role of chemoreception in lake trout (Buchinger et al., 2015; Foster, 1985; Wasylenko, Blanchfield, & Pyle, 2013; Zhang et al., 2001), but provoke a re‐evaluation of the data in the natural context of lake trout reproduction.

Populations within a species can experience different environments that favor different sensory strategies. One common example across taxa is the role different light environments have in shaping visual signals due to their transmission (Morrongiello, Bond, Crook, & Wong, 2010; Leal & Fleishman, 2003; ). Indeed, ecological diversity among lake trout populations creates several contexts that might favor alternative sensory strategies for selecting spawning habitats. Adaptive radiation in lake trout has resulted in various ecotypes that spawn in different habitats, including nearshore and offshore reefs, and rivers (Muir et al., 2012). While the ecologies of various ecotypes remain poorly understood (Muir, Hansen, Bronte, & Krueger, 2015), differences in spawning habitats conceivably translate into different degrees of overlap between when juveniles and adults occupy spawning locations. For example, juveniles in riverine populations may reside in streams until or past the time when adults begin spawning migrations (Goodier, 1981; Loftus, 1958). Juvenile residency at or near spawning habitat during the spawning season would provide a constantly replenished chemical cue for adults. Indeed, closely related anadromous char species likely select spawning habitat using the odor of stream‐resident juveniles (Nordeng, 1971). Lake trout in most populations have lost the anadromous phenotype (McLennan, 1994), but they detect juvenile odors with high sensitivity and specificity (Zhang et al., 2001; Zhang & Hara, 2009), deposit eggs over fresh feces in confined laboratory tests (Foster, 1985), and are attracted to juvenile odors (our results, Buchinger et al., 2015). Hence, juvenile odors are, or were, probably cues of historic reproductive success in some existing, or ancestral populations, of lake trout in which juveniles reside at or near spawning habitat during the spawning season. Unlike the examples of environmental constraints on transmission of visual signals (Morrongiello et al., 2010; Leal & Fleishman, 2003), lake trout might be a case in which differences in the habitat occupied across populations correspond to differences in behavior, and indirectly lead to shifts in sensory strategies.

Male odors may fill the role of juvenile odors in guiding spawning site selection for populations whose juveniles emigrate from spawning habitat before adults return (Buchinger et al., 2015). Males arrive at spawning reefs prior to females (Muir et al., 2012) and are conceivably well positioned to signal spawning aggregations to other adults. Indeed, substratum transferred from spawning reefs during the spawning season, possibly marked by males, appeared to attract spawning lake trout (Wasylenko et al., 2013), and both males and females are attracted to male odor (Buchinger et al., 2015). Interestingly, males do not behaviorally discriminate between male and juvenile odor. The indiscriminate behavioral response of males to male and juvenile odors indicates that preference for some component of male signal may have originated in another context and been exploited by males (*receiver bias*; Ryan & Cummings, 2013); males release an odor that appears to match the ancestral juvenile cue. Notably, the active components of both juvenile and adult odors are thought to be bile acids (Buchinger, Li, & Johnson, 2014; Zhang et al., 2001), whose release appears to be sexually dimorphic in spawning lake trout (Zhang, Brown, & Hara, 1996). Furthermore, other fish are believed to have evolved to use bile acids as pheromones through receiver biases (Buchinger, Wang, Li, & Johnson, 2013). While male odors may guide formation of aggregations on spawning reefs, additional cues must guide site selection for early arrivals.

Lake trout likely use a series of cues to select suitable spawning habitat. Most animals use multiple cues to select habitat, including unlearned cues that allow selection of novel habitats (Huijbers et al., 2012) and learned cues that allow natal homing (Walcott, 2005; Steck, Hansson, & Knaden, 2009; Lohmann, Putman, & Lohmann, 2008). Natal homing not only encourages local adaptation (Quinn & Dittman, 1990), but also serves as a mechanism of selecting habitat that previously supported successful reproduction (Greenwood, 1987). One of the best‐understood examples of natal homing is in Pacific salmon, which locate natal streams following stream‐specific chemical profiles learned as juveniles (Dittman & Quinn, 1996). In the context of anadromous spawning migrations, Bett and Hinch (2016) proposed that salmonids hierarchically respond to imprinted (1st), conspecific (2nd), and environmental (3rd) cues. Spawning lake trout also show site fidelity (Binder et al. 2016) and may return to natal spawning reefs (Bronte, Schram, Selgeby, & Swanson, 2002) following learned chemical profiles (Horrall, 1981). Site fidelity guided by learned odors may explain our observation that lake trout did not prefer constructed reefs treated with fresh fry feces, but rather returned to a spawning site used in previous years. However, the number of lake trout spawning on constructed reefs in Thunder Bay, and the number of fry produced on the reefs, increased steadily from 2012 to 2015, though these fish could not have been imprinted to the reefs constructed in 2010 (Marsden et al., 2016). Regardless of whether lake trout use imprinted cues to return to spawning areas, other cues are likely important for selecting specific spawning sites within a spawning area. Movement of lake trout among reefs within a spawning area (Marsden et al., 2016; Pinheiro, Stockwell, & Marsden, 2016) and rapid colonization of lakes in western North America (Martinez et al., 2009) indicates cues other than those learned by juveniles must be important during selection of specific spawning sites. We postulate that imprinted and conspecific cues act sequentially and that imprinted cues guide lake trout to a spawning area and conspecific odors, and other cues, facilitate aggregation on spawning reefs.

Our results underscore the importance of environmental constraints on the identity and function of cues (Endler, 1992). Temporal constraints on chemical cues used by terrestrial animals are well described; the efficacy of a cue depends, in part, on the rate at which it degrades or dissipates relative to the temporal scale of the social context (Alberts, 1992; Bossert & Wilson, 1963). The environmental constraints that act on aquatic chemical cues are equally important (Atema, 1995) but less‐often considered. Experiments that encompass the natural environmental context in which a chemical cue functions are crucial because those that do not can yield results that are difficult to interpret (Johnson & Li, 2010).

In summary, our evaluation of the hypothesis that lake trout use fry odors as cues of past reproductive success during selection of spawning habitat indicated that the efficacy of fry odors is temporally constrained and unlikely to facilitate spawning habitat selection in most lake trout populations. We postulate that odors learned as juveniles and male pheromone signals direct spawning site selection along with other nonolfactory cues. Lastly, we suggest that continued research on lake trout olfaction will yield an improved understanding of how chemical cues mediate important biological phenomena such as adaptive radiation (Muir et al., 2015) and may result in management implications for native and invasive populations (Muir, Krueger, Hansen, & Taylor, 2013).

## CONFLICT OF INTERESTS

The authors declare that they have no conflict of interest.

## AUTHOR CONTRIBUTIONS

TJB participated in all aspects of developing and writing the manuscript. JEM and TRB contributed to all aspects of the field test. MH contributed to EOG recordings and UB quantified bile acids in water samples. KL and JEH designed and prepared odor treatments for behavioral tests. CCK contributed to the field test. WL and NSJ contributed to all aspects of the study. All authors participated in writing the manuscript and approved the final version.
